# How far on the road? The role of family medicine/general practice in 10 Central and Eastern European countries: A mixed-method study

**DOI:** 10.1080/13814788.2025.2594292

**Published:** 2025-12-17

**Authors:** Marek Oleszczyk, Aleksander Stepanovič, Norbert Král, Bohumil Seifert, Igor Švab, Janusz Krzysztoń, Natalia Jagiełła, Adam Windak

**Affiliations:** ^a^ Department of Family Medicine, Jagiellonian University Medical College, Krakow, Poland; ^b^ Department of Family Medicine, Medical Faculty University, Ljubljana, Slovenia; ^c^ Health Care Center Škofja Loka, Primary Health Care of Gorenjska region, Škofja Loka, Slovenia; ^d^ Department of Family Medicine, 1st Medical Faculty, Charles University, Prague, Czech Republic; ^e^ Family Physicians’ Group Practice Ltd, Krakow, Poland

**Keywords:** Family medicine, general practice, primary care, Central and Eastern Europe

## Abstract

**Introduction:**

Central and Eastern European (CEE) countries began healthcare reforms in the late twentieth century, adopting Family Medicine/General Practice (FM/GP) models. The FATMEE (Family Medicine After Transformation in Middle and Eastern Europe) study in 2012 found this process advanced but incomplete. This study (FATMEE-2) examines current FM/GP development in the CEE region that follows recent social changes and healthcare challenges.

**Methods:**

A mixed-methods approach combined literature and dataset review with a Key Informant-based survey using the updated FATMEE questionnaire, exploring the FM/GP role in primary care.

**Results:**

FM/GP is recognised as a separate medical speciality in all countries with robust legal frameworks. Care comprehensiveness varies, with some systems maintaining separate paediatric and adult services, and a lack of gynaecology and obstetrics services in many. Weighted capitation remains the dominant funding model, supplemented by pay-for-performance and fee-for-service schemes. Electronic medical records and teleconsultations are common. Compared to the previous FATMEE study, changes include increased use of digital tools and diversified financing. Primary care structure and professional roles changed little.

**Conclusion:**

While the legal and technological foundations of family medicine in CEE countries have strengthened, comprehensiveness and service integration have limitations. There is a visible progress in infrastructure and digitalisation, but the structural and organisational challenges identified in 2012 largely remain. This may indicate that sustained political commitment and systemic reform – beyond legal acknowledgement and technological improvements – are essential for successful transformation. However, the examples of Estonia and Slovenia prove that under a supportive policy, such a transformation is achievable.

## Introduction

Along with the political changes at the end of the twentieth century, post-communist Central and Eastern European (CEE) countries sought their way to adapt various areas of socio-economic life to Western European standards and norms. The changes included reforms of the mostly ineffective healthcare systems, with the transformation of primary care (PC) and the introduction of Family Physicians/General Practitioners (FPs/GPs) as the main actors in PC, with broad competencies, continuity of care, and a gate-keeping role [1–4].

Studies from the early twenty-first century reported that the introduction of FM/GP was nearly universal in CEE countries, although incomplete [5,6]. This indicated that additional efforts are needed to strengthen the role of FM/GPs in the provision of primary care (PC) [5,6]. Recent social and political challenges, including the COVID-19 pandemic, stressed European healthcare systems. FM/GP had to adapt to new conditions and is still redefining its role in the changing environment, as reflected in the updated WONCA-Europe definition [7–11]. However, a comprehensive picture of FM/GP in CEE countries is not available. The Family Medicine After Transformation in Middle and Eastern Europe 2nd Edition (FATMEE-2) project aims to examine the advancements in FM/GP development in CEE countries. It is based on the results of the 2012 FATMEE project [12,13].

The aim of the FATMEE-2 study is specifically to assess the state of development of FM/GP in CEE countries as a medical specialty and as an academic and scientific discipline. In this paper, we aim to explore the place of FM/GP and the practitioners in the health care system, specifically in PC. We approach this by answering the following questions:

- What is the legal status of FM/GP in the health care system of the CEE countries?
- How are PC and FM/GP financed in the CEE countries?
- What is the scope of services of FPs/GPs in the PC?

## Methods

A consortium of three universities conducted the study: Charles University in Prague, Jagiellonian University in Kraków, and the University of Ljubljana. FATMEE-2 aimed to gather data from all 21 CEE countries. Ukraine, Belarus, and Russia were excluded due to their involvement in the ongoing war.

Data were collected using a mixed-methodology approach, employing an opportunistic literature review, dataset review, and a key Informant survey.

### Key-informant survey

The informants were selected from prominent experts in FM/GP, usually national representatives of WONCA-Europe member organisations or their networks.

The data were collected between June 2022 and December 2023. Key informants gave informed consent before completing the survey. Only countries with complete responses from at least two experts were included. In cases of inconsistent answers, informants or other local experts were contacted for clarification. Details of the survey and data collection are presented according to the CHERRIES protocol in Supplemental Material 1.

### Literature and datasets review

The data search covered the period from 2012 to 2021. The search included Medline-PubMed, Embase, Google Scholar, and datasets such as OECD Health Stats, WHO Observatory’s Healthcare in Transition, and WHO Health at Glance report series [14–16].

### Questionnaire

Based on the review results, the original FATMEE questionnaire was modified. All FATMEE-2 consortium partners reviewed the draft. The final FATMEE-2 questionnaire, consisting of 109 items, was created, covering four major topics: (1) Role in the Health Care System, (2) Quality Assurance and Improvement, (3) Medical Education, and (4) Research and Scientific Activities. The data analysed in this publication are based on responses to questions in part (1), Role in the Health Care System, consisting of 61 items. The questionnaire was adapted for each country using local data, allowing Key Informants (KIs) to accept, reject, or correct predefined answers. An example of the questionnaire is provided in Supplemental Material 2.

The Research Electronic Data Capture (REDcap) was used to collect data, which was then exported to a common MS Excel dataset. Necessary calculations were performed using MS Excel functionalities.

### Ethical issues

The study has been approved by the Bioethical Committee of the Jagiellonian University in Kraków, Poland (opinion No. 1072.6120.39.2021).

## Results

### Countries included in the analysis, along with their characteristics

Of the 18 approached countries, the complete set of responses of two national key informants was obtained in ten (55%) of them: Croatia, the Czech Republic, Estonia, Montenegro, North Macedonia, Poland, Romania, Serbia, Slovakia, and Slovenia. Seven of the FATMEE-2 countries were included in the original FATMEE study (Croatia, Czech Republic, Estonia, Poland, Romania, Slovakia and Slovenia).

### Characteristics of the key informants

Of 20 key informants, 11 (55%) were female; the mean age was 43.5 years (min. = 35, max. = 64). All the key informants were physicians with specialisation in FM/GP. Seven of them (35%) had the title of Professor, eight (40%) had a PhD degree, 2 (10%) had – Master of Science, and two (10%), Master’s in Public Health. Primary care facilities were ranked as the main working place by 11 (55%), university or medical faculty by six (30%), and both by three (15%).

### Legal status of family medicine/general practice in the healthcare system

Parliamentary acts and/or ministerial decrees regulate the place of FM/GP in HCS in all studied countries, except North Macedonia, which has no such regulations.

In seven countries (the Czech Republic, Croatia, Poland, Romania, Serbia, Slovakia, and Slovenia), the competencies of FPs/GPs were described in legal documents, whereas in Estonia and Montenegro, such documents are not present. The central government regulates competencies in the Czech Republic, Poland, Romania and Serbia; insurance companies – in the Czech Republic, Romania, Slovenia, and Serbia; scientific professional colleges or associations – in the Czech Republic, Romania and Slovenia; and the medical chamber in Slovenia. Non-governmental, independent organisations act by issuing standards and guidelines for PC, providing accreditation programs, and representing FPs/GPs in negotiations with stakeholders and insurance companies.

FM/GP is recognised as a separate speciality in all countries and has legal status equal to other medical disciplines.

### Financing of primary care

In all participating countries, the central/federal government and health insurance companies are the authorities responsible for financing PC services. The local government also plays an essential organisational role in the Czech Republic, Croatia, Poland, Slovakia, and Slovenia. Medical colleges/associations (Estonia, Slovakia, and Slovenia) or medical chambers (in the Czech Republic) also regulate PC services.

**Figure 1.**
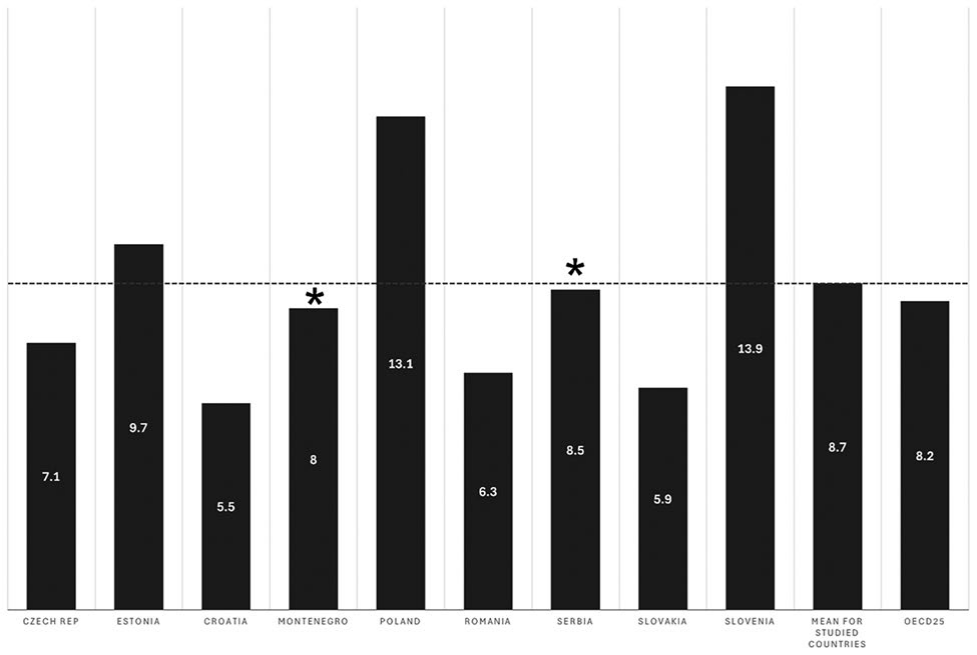
Primary health care expenditures as the ratio of all health care expenditures; based on OECD Health Statistics (countries marked with• - data from key informants; data from North Macedonia unavailable).

The percentage of health care expenditures dedicated to PC varies in the studied countries, from 5.5 in Croatia to 13.1 in Slovenia, with a mean of 8.7%. This ratio is higher than the mean for the OECD in Estonia, Poland, Serbia and Slovenia. Details are presented in Figure 1.

All countries finance FM/GP services using mixed systems. The predominant payment method is capitation; in some countries, it is a weighted capitation fee (e.g. for age or chronic disease). Table 1 presents details about the payment methods used in different countries.

**Table 1.** Financing of the family medicine/general practice services.

	Fixed budget	Capitation fee	FFS	PFP	Other
CZ		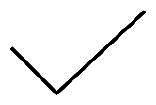	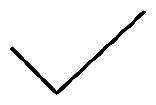	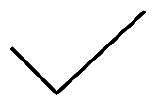	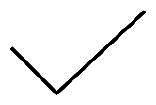
EE		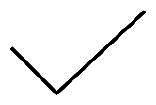 *	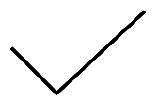	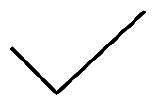	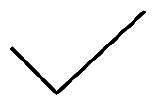
Croatia	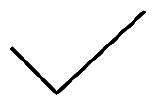	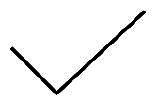		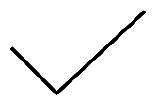	
ME		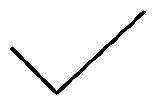		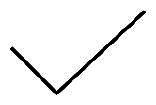	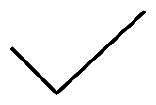
MK		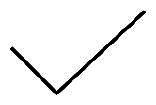			
PL		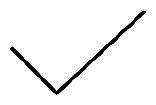 *	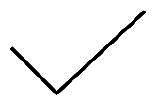	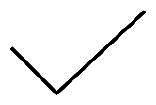	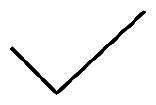
RO		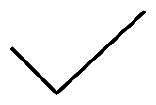	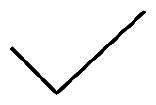		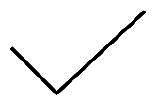
RS	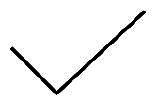	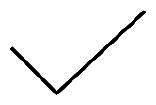			
SK		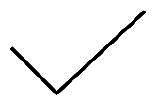	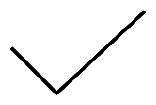		
SI	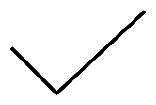	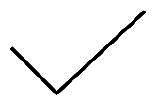 *	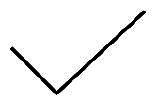	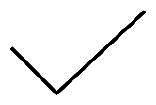	

FFS, fee for service; PFP, pay per performance; CZ, Czech Republic; EE, Estonia; HR, Croatia; ME, Montenegro; MK, North Macedonia; PL, Poland; RO, Romania; RS, Serbia; SK, Slovakia; SI, Slovenia.

* Weighted capitation fee.

### Role of family physicians/general practitioners in primary care

Only in Estonia do FPs/GPs exclusively provide PC services; in all other countries, physicians of different backgrounds operate in the PC setting (Table 2). Except for the Czech Republic and Slovakia, physicians without any specialisation can work in PC (although under specific conditions, such as a subcontractor of a licenced FP/GP).

**Table 2.** Physicians providing services in primary care.

	FP/GP	Internist	Pediatrician	O&G specialist	Occupational medicine specialist	Other specialist	Physicians without specialisation
CZ	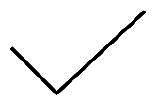		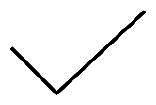	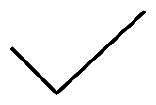		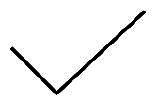	
EE	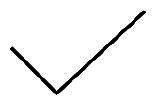						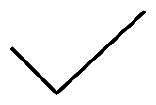
HR	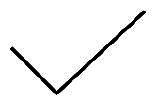		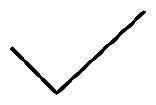	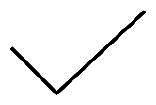	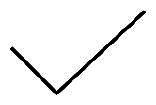	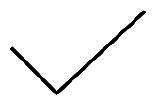	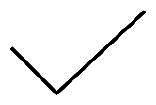
ME	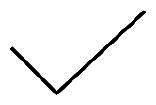	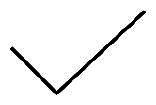	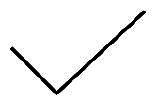	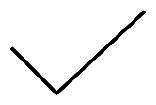	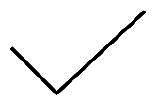	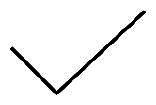	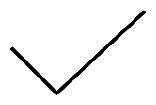
MK	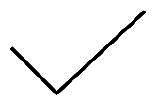		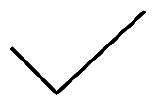	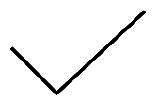	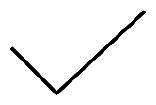		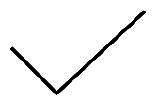
PL	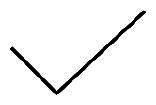	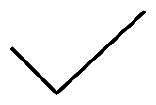	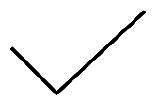			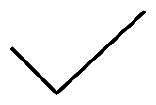	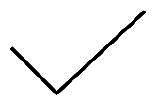
RO	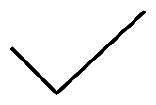						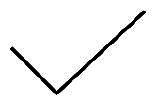
RS	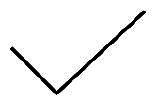	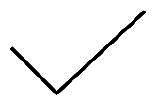	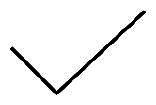	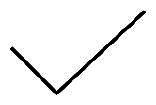	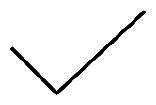	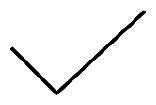	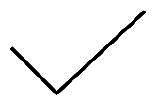
SK	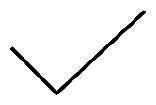	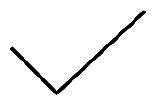	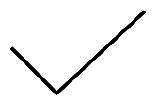	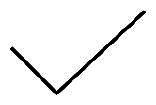			
SI	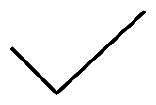		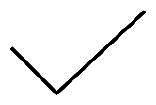	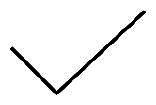	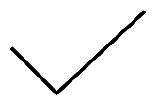		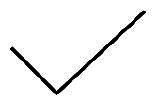

FP/GP: Family physician/general practitioner; O&G: Obstetrics and gynaecology; CZ: Czech Republic; EE: Estonia; HR: Croatia; ME, Montenegro; MK North Macedonia; PL Poland; RO Romania; RS Serbia; SK Slovakia; SI Slovenia.

**Figure 2.**
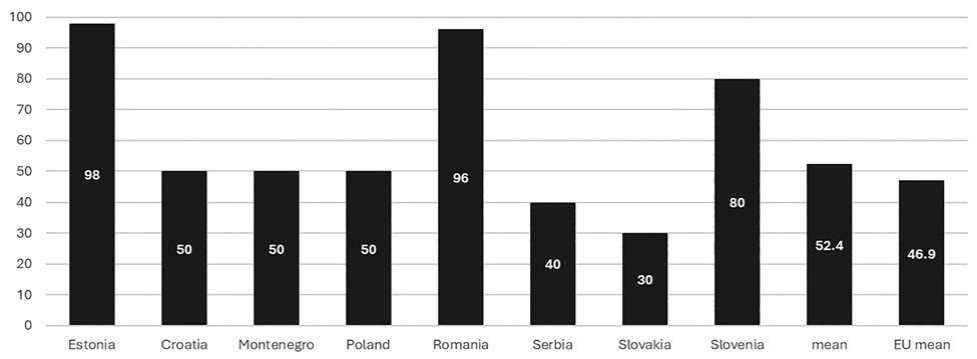
Ratio of family physicians/general practitioners in all primary health care physicians (missing countries- data not available).

The median age of FPs/GPs is nearly 50 (ranging from 47.5 in Montenegro to 59 in North Macedonia). In the Czech Republic, Estonia, Poland, Romania, Slovakia, and Slovenia, physicians must meet special entry conditions (e.g. a specialty in FM/GP, internal medicine or paediatrics) to work in PC. There are no such entry conditions in Croatia, Montenegro, North Macedonia, and Serbia. The ratio of FM/GP specialists to all PC physicians is shown in Figure 2.

#### Employment status

In the seven studied countries, FPs/GPs work either as independent contractors or the employees of the independent contractors (Czech Republic, Estonia, Croatia, North Macedonia, Poland, Serbia, Slovakia, Slovenia). However, in Croatia, Montenegro, Romania, Serbia, and Slovenia, the central or local government can also employ them directly.

#### Patients’ lists

**Figure 3.**
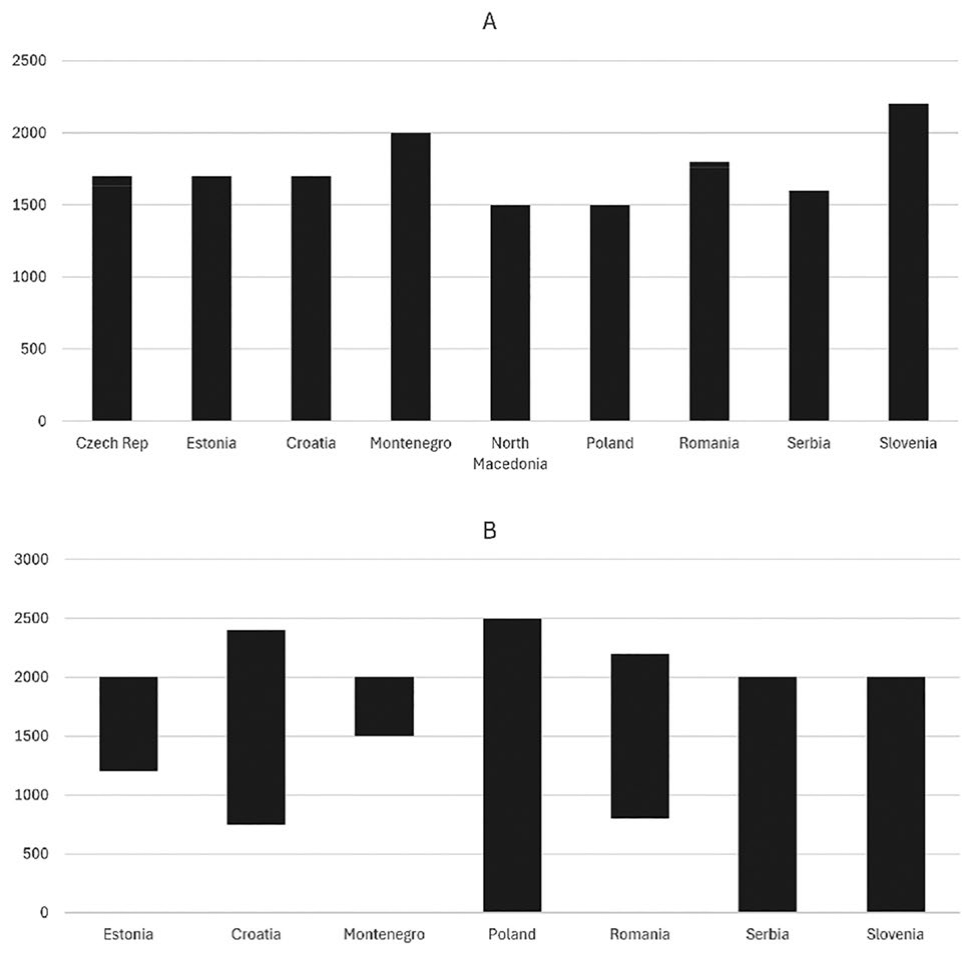
The average number of patients on the list of family physicians/general practitioners (A) and minimal and maximal size of the lists of patients (B) (missing countries - data unavailable or no regulations do not apply).

All countries have FP/GP patient lists, although six countries (Estonia, Croatia, Montenegro, Poland, Romania, Serbia, and Slovenia) set limits on list sizes. The Czech Republic has unenforced regulations on the size of the patients’ list, while North Macedonia and Slovakia have none. In Estonia, exceeding 2,000 patients requires hiring an additional doctor; fewer than 1,200 patients reduce the base fee (except in remote areas). Poland’s default maximal list accepted by the national health insurance company is 2,500 patients, but in selected cases (e.g. in remote practices), a larger list can be accepted. Romania reduces fees for patients beyond the cap, while Slovenia offers bonuses for larger lists (however, to a limit of 140% of the average list size, above which the fees are also reduced). The average patients’ list size and limits are presented in Figure 3.

Although solo practices are predominant or frequent service organisations in most studied countries, there are many differences between the health care systems. Details are presented in Supplemental Material 3.

In some participating countries, the working hours of FPs/GPs and, in some cases, the time spent consulting patients are regulated. Details are presented in Supplemental Materials 4.

### Range and forms of service provision

In Estonia, Croatia, Montenegro, Poland, Romania, and Slovenia, patients must be registered with an FP/GP to access services, while in the remaining four countries, enrolling the patients on the list is recommended, however, not obligatory. Services provided by FP/GPs in CCE countries are outlined in Table 3. In the Czech Republic, they also offer occupational medicine, preventive health consultations, first aid education, and collaborate with local authorities for special needs patients. Service formats are similar across countries, including office visits, home visits, and telemedicine (except in North Macedonia). Social care assessments are common, except in North Macedonia and Romania. Patient group sessions are offered only in Croatia, Montenegro, and Slovenia.

**Table 3.** Services provided by family physicians/general practitioners.

	Curative care for children and adolescents	Curative care for adults	Pregnancy and postnatal care	Children surveillance and preventive care (including vaccination)	Adults screening and preventive programs (including vaccination)	Assessment/medical certification for social services and social insurance purposes	Occupational medicine
**CZ**	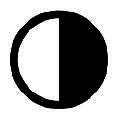	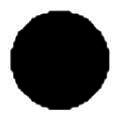	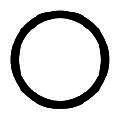	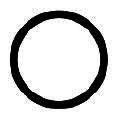	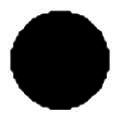	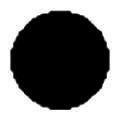	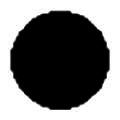
**EE**	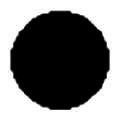	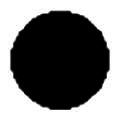	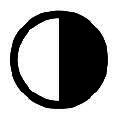	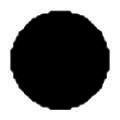	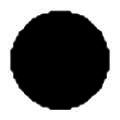	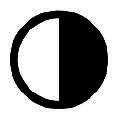	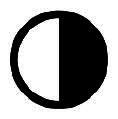
**HR**	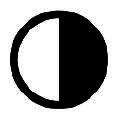	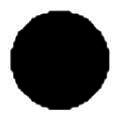	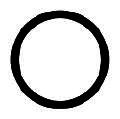	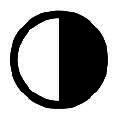	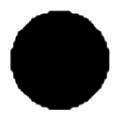	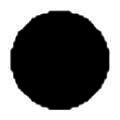	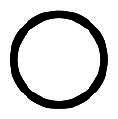
**ME**	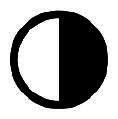	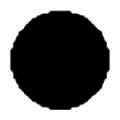	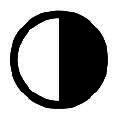	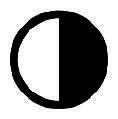	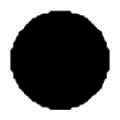	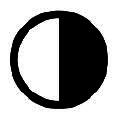	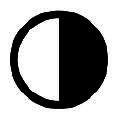
**MK**	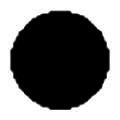	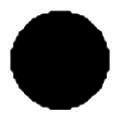	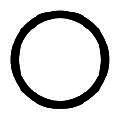	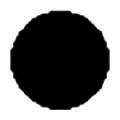	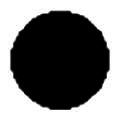	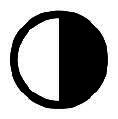	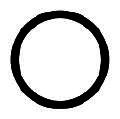
**PL**	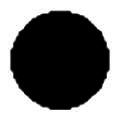	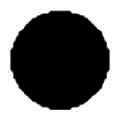	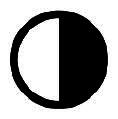	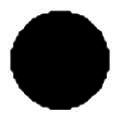	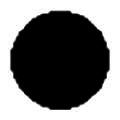	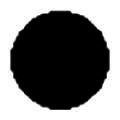	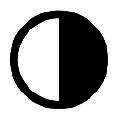
**RO**	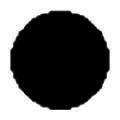	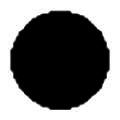	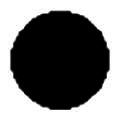	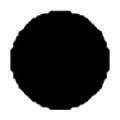	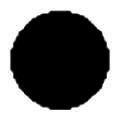	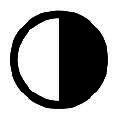	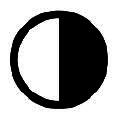
**RS**	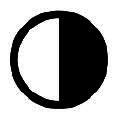	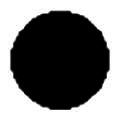	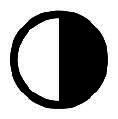	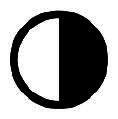	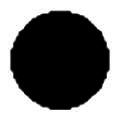	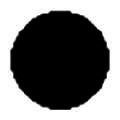	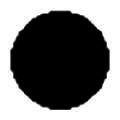
**SK**	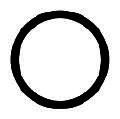	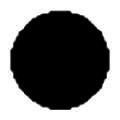	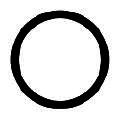	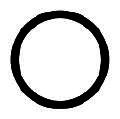	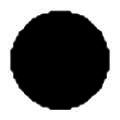	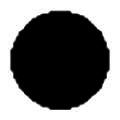	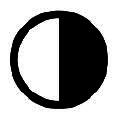
**SI**	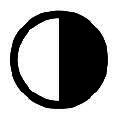	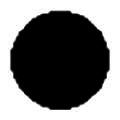	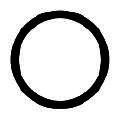	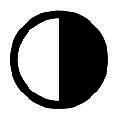	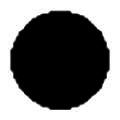	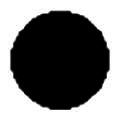	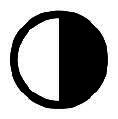

CZ: Czechia; EE: Estonia; HR: Croatia; ME: Montenegro; MK: North Macedonia; PL: Poland; RO: Romania; RS: Serbia; SK: Slovakia; SI: Slovenia.

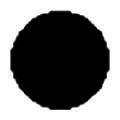
Always 
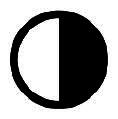
Sometimes 
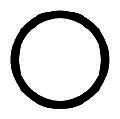
Never.

### Gate-keeping

In all studied countries, FPs/GPs are gatekeepers in the public health care system; however, in the case of the Czech Republic, Estonia, Poland, Romania, Slovakia, and Slovenia, only partially. Some specialist services are available without a referral from the primary care physician. Table 4 summarises the range of gatekeeping.

**Table 4.** Specialities accessible without a referral from family physicians/general practitioners.

	O&G specialist	Paediatrician	Internist	Ophthalmologist	ENT specialist	Oncologist	Dermatologist	Surgeon	Dentist	Psychiatrist
CZ	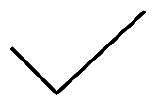	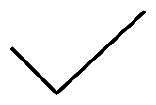	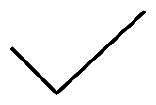	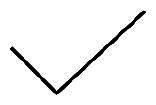	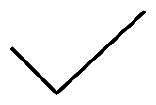		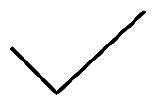	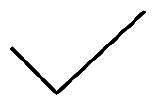	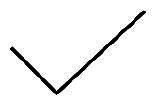	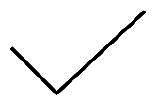
EE	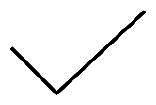			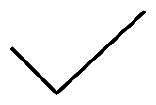					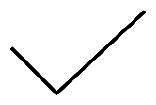	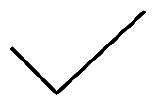
HR	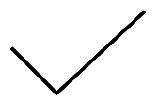	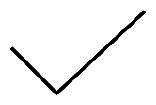								
ME	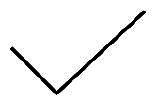	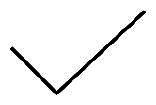								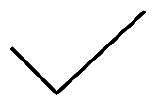
MK	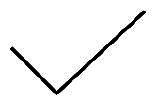	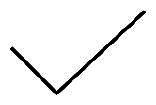								
PL	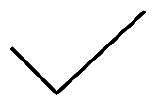					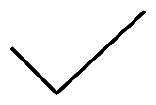			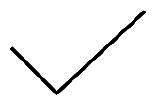	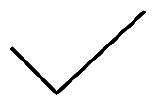
RO	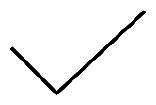	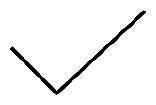				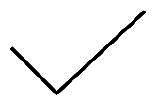			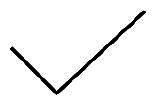	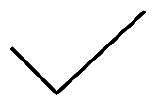
RS	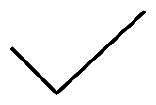	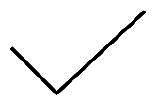							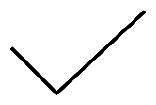	
SK	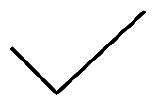	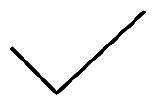					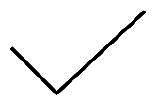		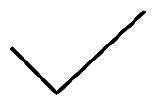	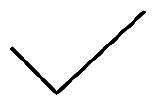
SI	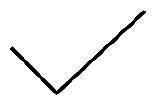	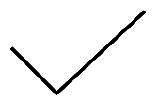		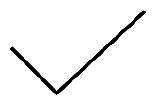					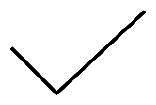	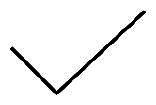

ENT: Laryngologist; O&G: Obstetrics & gynaecology; CZ: Czech Rep; EE: Estonia; HR: Croatia; ME: Montenegro; MK: North Macedonia; PL: Poland; RO: Romania; RS: Serbia; SK: Slovakia; SI: Slovenia.

### Out-of-hours care

FPs/GPs are obliged to provide out-of-hours care only in Croatia, Montenegro, Serbia and Slovenia. In the Czech Republic, Estonia, Romania, and Slovakia, FPs/GPs can be voluntarily involved. FPs/GPs do not organise out-of-hours services in North Macedonia and Poland. Currently, no deputising services are hired by PC doctors to provide out-of-hours care for their patients. In Estonia, family doctors can extend working hours with a special contract (to work >8 h per day or on Saturday). Types of out-of-hours services are presented in Supplemental Materials 5.

## Discussion

### Main findings

Our study aimed to investigate and compare information regarding key elements of PC and FM/GP roles in European countries with similar experiences and historical and political backgrounds. In the studied CEE countries, FM/GP has a solid legal foundation. Central/federal governments and health insurance companies, with some local government tasks, are the main organisers and payers of PC services. Capitation fee payment remains the primary payment method for PC; however, the income structure is now more diverse. Most studied countries have not provided FM/GP specialists to fulfil all PC services. Obstetrics and gynaecology services are mostly not provided by FPs/GPs, and in some cases, paediatric and adult care remain separated.

### Strengths and limitations

Unlike studies that focus on specific topics or conditions, FATMEE-2 offers a broader overview of FM/GP development, shedding light on the current status of PC in the region. An important limitation in the generalisability of the findings is that, in the end, we were able to analyse data from only 10 of 17 approached countries. The key informant methodology provides access to expert knowledge, including legal regulations, often absent in medical literature, and helps clarify complex systems such as national healthcare. However, there is always some risk of bias and an impact on results and their interpretation caused by key-informant selection.

The lack of comprehensive and good-quality datasets in many areas makes the precise measurement of some indicators difficult. Another limitation is that we are reporting on constantly evolving policies. Nevertheless, the main trends in health policies in the region can still be identified as valid. It is worth noting that, although the CEE countries have a similar historical background due to their location ‘behind the Iron Curtain’, treating them as a coherent whole carries the risk of simplification. The differences between the countries that were part of the Soviet Union (e.g. Estonia), close satellites of that power (e.g. Poland, Czechoslovakia), and the countries of the former Yugoslavia (e.g. Croatia or Slovenia) in various aspects of socio-economic life were considerable.

### Interpretation of existing literature

#### Comparison with the FATMEE 1st edition results

Since the original FATMEE study, FM/GP has gained a solid legal basis and is now an integral part of PC systems in all studied countries. Estonia remains the only country with exclusive family medicine in primary care, while Slovenia has become a co-leader due to its successful implementation of reforms. Both countries continue to show innovation in payment systems and technology adoption. Technological advancements occurred across all countries – even those with limited structural progress, such as Poland and the Czech Republic, have achieved significant EMR adoption and telemedicine integration. Most countries face some structural stagnation, e.g. Poland’s planned 2017 transition to FM as an exclusive PC specialty remains incomplete, and the mixed specialisation model persists in most countries despite a decade of potential reform. Payment systems are more sophisticated everywhere, with all countries moving from basic capitation to complex mixed systems that include performance incentives and demographic adjustments. Service scope has expanded modestly; telemedicine is nearly universal, and some countries offer patient group sessions. However, obstetrics and gynaecology services are still largely excluded from the FM/GP scope. A more detailed comparison of the FATMEE-1 and 2 are presented in Supplemental Material 6.

#### Comparison with other studies and reports

In the global view of FM/GP development, the CEE countries are regarded as the second, already mature generation [17]. The reports from the CEE region present Slovenia [18,19] and Estonia [20] as examples of a successful PC transition and innovation, long-term consistency in achieving PC reform goals, in combination with skilful use of financial incentives for PC physicians, is debated as the foundation for success. In the Czech Republic, a better residency program and remuneration system improved the status of FPs/GPs. The situation in other countries is perceived with less enthusiasm. In Bosnia and Herzegovina, the FM/GP development plan is evaluated as partially completed, primarily due to a lack of sharing common goals with stakeholders [21]. However, the PC reform model based on FM/GP is still perceived as practical and attractive; for example, Kosovo, the newly independent territory in the region, decided to follow in the footsteps of the neighbouring countries [22]. On the other hand, external factors like the COVID-19 pandemic and war are negative stressors that test the resilience of the PC in Ukraine, forcing a change of priorities and plans [23]. Challenges in PC and FM/GP are reported globally, not limited to the CEE region. Other European high-income countries, such as Germany or Greece, face barriers FM/GP development [24], or Greece [25]. In the United States, underinvestment, IT burdens, and workforce shortages hinder progress [26] All the time, FM/GP must adapt to changing conditions as the speciality is meant to serve in PC [7,27]. Overall, PHC reform outcomes depend on multiple interrelated factors and require context-specific adaptation. As one review concludes, the PC reform outcomes depend on a combination of heterogeneous factors that must be taken into account [28].

### Implications (clinical practice, education, policy)

The study highlights the need to enhance FM/GP comprehensiveness. Additional research on physician and patient opinions could help address gaps in O&G procedures and the division between paediatric and adult care in some countries. Medical graduates’ career choices impact FM/GP training and workforce growth. Strategies to make FM/GP more appealing and long-term national policies supporting PC development are essential. Collaboration among national and international organisations, with WHO, EU, or WONCA-Europe guidance, can help shape future PC discussions in CEE countries.

## Conclusion

Implementing FM/GPs into PC in CEE is ongoing. There is a significant variability among the countries. Estonia and Slovenia are perceived as outstanding leaders. FM/GPs have established a place in the HCS, but their gate-keeping role differs in the countries studied. E-health and telemedicine play now important role in PC provision. EMR and digital information processing are universally present in PC. The care provided by FPs/GPs still lacks some comprehensiveness – with some exceptions, obstetrics and gynaecology are not routinely part of the FM/GP tasks, and in some countries there is still a separation between paediatric and adult care. The reasons for this could be historical or cultural; however, with the existing data, it cannot be concluded. The data shows that, despite major progress in legal and technological areas, the core challenge identified in 2012 – creating comprehensive, integrated family medicine models – still largely persists after twelve years. This suggests that achieving sustainable healthcare transformation depends on ongoing political commitment and significant system changes, not merely legal acknowledgment and technological improvements.

## Supplementary Material

Supplemental Material
