# Supplementary material for: How far on the road? The role of family medicine/general practice in 10 Central and Eastern European countries: A mixed-method study
Source: Eur J Gen Pract. 2025 Dec 17;31(1):2594292. doi: 10.1080/13814788.2025.2594292 (PMC12713223; doi:10.1080/13814788.2025.2594292)
Supplement: Supplemental Material [file IGEN_A_2594292_SM2077.zip › IGEN_A_2594292_suppl_data/ejgp-2025-0118-File006.docx]

**Supplemental material 2. Example of the FATMEE-2 questionnaire (country-specific version for Poland)**

**Role in the Health Care System**

Is there any official policy document presenting the current role of Family Medicine/General Practice in your country? Yes/No

Yes based on Poland: Health care system review (2019), p.95

You stated that there is formal acknowledgment of

You stated there is an official policy document

parliamentary law

presenting the current role of Family Medicine/General

ministerial decree

Practice in your country.

document of Medical Chamber

What kind of document is it?

document of a professional association

scientific paper

other

Is there any official policy document presenting the

Yes

future development of Family Medicine/General Practice

No

in your country?

You stated there is an official policy document

parliamentary law

presenting the future development of Family

ministerial decree

Medicine/General Practice in your country. What kind

document of Medical Chamber

of document is it?

document of professional association

scientific paper

other

Are the competencies of FPs/GPs clearly described?

Yes

No

)

(

FPs/GPs - Family Physicians/General Practitioners

You stated that the competencies of FPs/GPs are

central/federal government

clearly described? If so, at which level?

insurance company(ies)

Please choose all applicable.

local/regional authorities

scientific [professional] college or association

other medical organization (e.g. medical chamber)

other

FPs/GPs - Family Physicians/General Practitioners

)

(

Who is responsible for organization of FPs/GPs

central/federal government

services?

insurance company(ies)

Please choose all applicable.

local/regional authorities

scientific [professional] college or association

other medical organization (e.g. medical chamber)

other

(

FPs/GPs - Family Physicians/General Practitioners

)

Is specialty in Family Medicine/General Practice

Yes

formally recognized?

No

Yes

Family Medicine/General Practice as separate medical

No

specialty. If so, has it the same formal status as other specialties (e.g. internal medicine or general surgery)?

Are there any special requirements for medical doctors Yes

to work in primary care (e.g. specialty in Family No

Medicine/Genral Practice; specialty in Pediatrics etc.)

What sort of physicians provide services in primary

family physician/general practitioner

care in your country?

internal medicine specialist

Please choose all applicable.

pediatrician

obstetrician/gynecologist

occupational medicine specialist

other specialist

physicians without specialisation

| You stated, that there are OTHER than listed medical specialties in Primary Care. Please name them. | __________________________________________ |
| --- | --- |
| What is the ratio of physicians with a specialty in  Family Medicine/General Practice in all primary care | __________________________________ |
| physicians physicians ((range: 0 - 100)) | |

What is the occupational formal status of FPs/GPs?state employees local government

Choose all applicable. employees independent contractors

employees of contractors other

(FPs/GPs - Family Phsyicians/General Practitioners)

| What is total expenditure on primary care as percantage (%) of total public expenditure on health | __________________________________ |
| --- | --- |
| (exluding dental care)?    13.5 %    based on OECD Healt at Glance 2021 | ((range: 0 - 100)) |
| What is the number of consultations in ambulatory care per capita per year?  7.7  Based on OECD Healt at Glance 2021 | __________________________________ |

| If so, are the patients obliged to enroll on the list to use PC services?  What is the average number of the patients on the | (FPs/GPs - Family Physicians/General Practitioners) |
| --- | --- |
| list?  You stated that FPs/GPs have their lists of patients. | __________________________________ |
| If so, is there a minimum and/or maximum number of the patients on the list?  You stated that there is a minium and/or maximum | (FPs/GPs - Family Physicians/General Practitioners) |
| number of the patients on the list. Please name the minium.  You stated that there is a minium and/or maximum | __________________________________ |
| number of the patients on the list. Please name the maximum. | __________________________________ |

You stated that FPs/GPs have their lists of patients.

Yes

No

Do FPs/GPs have their patient lists?

Yes

No

(

FPs/GPs - Family Physicians/General Practitioners

)

Yes

No

No, but it is preferred

Are there any measures (e.g. in payment) employed to

influence the maximum or minimum size of the list?

Yes

No

You stated there are mechanisms to maintan minimum and/or maximum on the list of the patients.

please describe them shortly.

__________________________________________

How are the primary care services financed?

Please choose all applicable.

fixed budget

capitation fee

capitation fee with wages

fee-for-service

pay-per-performance

other

How is the FPs/GPs work paid?

Please choose all applicable.

salary

income from the contract (with

authoreties/health insurance company)

directly by patients (out of pocket)

)

FPs/GPs - Family Physicians/General Practitioners

(

Are the FPs/GPs gatekeepers in the health care system?

Yes

Yes, partially

Not at all

(

FPs/GPs - Family Physicians/General Practitioners

)

Which of the following specialties are accessible

without a referral from the FPs/GPs in the public

health care system (predominant health insurance

framework)?

Please choose all applicable.

Gynaecologist /obstetrician

Pediatrician

Internist

Ophthalmologist

ENT specialist

Oncologist

Dermatologist

Surgeon

Dentist

Psychiatrist

Palliative care specialist

Other

(

FPs/GPs - Family Physicians/General Practitioners

)

Are the daily working hours of Family

Physicians/General Practitioners regulated?

Yes

No

| You stated that daily working hours of FPs/GPs are regulated. Please describe briefly, what are the regulations? | __________________________________________ |
| --- | --- |
| You stated that daily working hours of FPs/GPs are regulated. Please write how many hours it is daily. | __________________________________ |
| You stated that daily working hours of FPs/GPs are regulated. Please write how many hours daily they have to spend in the direct contact with the patients (in the office/home visits) | __________________________________ |

Is out-of-hour care (nights, weekends, holidays & festivals) part of the FPs/GPs responsibilities? yes, obligatory yes, voluntary

no, not at all

How is out-of-hour primary care organized? Please, choose all applicable.

(FPs/GPs - Family Physicians/General Practitioners)

Rota of duties shared by several practices

(physician) Deputizing service hired

Emergency service Hospital-based centers Special separate service organized by specialized companies

Other (please describe briefly)

You stated, that there is other than listed

organization of out-of-hour care. Please describe

briefly. __________________________________________

**What are the services that are typically provided by Family Physicians/General Practitioners?**

always sometimes never

Curative care for children and adolescents

Curative care for adults

Pregnancy and postnatal care

Children surveillance and preventive care [including vaccination]

Adults screening and preventive programs [including vaccination]

Assessment/medical certification

for social services/social insurance purposes

Occupational medicine

**What are the forms of services provided by the family physician**

Yes

No

Office consultation

Home visits

Telemedicine (telephone/ video

consultations/remote

monitoring)

Group sessions

Assessment for social services

Other

You stated, that there are other than listed forms of

providing FPs/GPs services.

| Please, describe briefly | __________________________________________ |
| --- | --- |

How are the medical records/medical documentation EMR exclusively traditional processed in primary care? hardcopy/paper medical records

mixed - EMR & traditional hardcopy/paper

medial records

(EMR - electronic medical records)

**How the informative/computerized systems are used in the Primary Care Practicies?**

In (nearly) all in majority of in half of the in some of the rarely or never practices practicies/ practities practicies

Administrative purposes

Clinical care (electronic medical records, referrals, prescriptions etc.)

Other purposes

| You pointed that there are other than listed purposes |  |
| --- | --- |
| of computerized systems in primary care practities. |  |
| Please, describe them briefly | __________________________________________ |

**Rank the following organizational forms of primary care practicies**

**)**

**FPs/GPs -Family Physicians/General Practitioners**

**(**

predominant

frequent

rare

not existing

Single-handed (solo)

Single-handed (solo) as part of

chains/cooperatives

Small group practicies of less

than 6 FPs/GPs

Larger group practicies/health

centers with 6 or more FPs/GPs

Health centers with FPs/GPs and

other specialists

| What is medium age of practicing FPs/GPs in your country | __________________________________  (FPs/GPs - Family Physicians/General Practitioners) |
| --- | --- |
| What is median of age of practicin FPs/GPs in your country? | __________________________________  (FPs/GPs - Family Physicians/General Practitioners) |

Is the WONCA definition of family medicine recognizedYes

and implemented by health authorities and/or healthNo

policymakers?I do not know

(WONCA - World Organization of Family Doctors)

The European Definition of GP / FM

| **Quality Assurance and Improvement** |  |
| --- | --- |
| Please name important voluntary mechanisms in your country to maintain and improve the quality of care provided in primary care (e.g. clinical guidelines, voluntary peer-review mechanisms). | __________________________________________ |

Have evidence-based clinical guidelines been developed

Yes

or adapted for specific use by FPs/GPs?

No

)

(

FPs/GPs - Family Physicians/General Practitioners

Is there any system of registration, reporting and

Yes

monitoring medical errors in primary care?

No

Is there any accreditation system of FPs/GPs

obligatory

practicies in the country?

voluntary

does not exist

(

FPs/GPs - Family Physicians/General Practitioners

)

You stated there is an accreditation system for

governmental body

Primary Care practicies. Who is responsible for it?

health insurance company

Please choose all applicable

FM-GP association or college

other non-government organization

other

What ratio of primary care practities is accredited?

__________________________________

Are there any incentives to undergo the accreditation

process?

Choose all applicable

financial (e.g. higher fee, lower taxes)

higher chance to get the contract with

health insurance company

internal motivation only

other (please describe briefly)]

Do primary care practicies undergo any other common

accreditation/certification (e.g. ISO)?

Yes

No

You stated that primary care practicies undergo some

other accreditation/certification procedures.

Please name them (and briefly decribe if necessary). __________________________________________

Do peer-review groups exist and are active in your

country?

Yes

No

You stated that peer-review groups exist and are

active in your country. __________________________________

What ratio of FPs/GPs is involved? ((range 0-100))

Are there any incentives to participate in the

peer-review goups?

financial (e.g. higher fee, lower taxes)

higher chance to get the contract with

health insurance company

internal motivation only

other (please describe briefly)]

| **Teaching of Family Medicine/General Practice and Academic Development** | |
| --- | --- |
| How many Medical Universities/Academies are there in your country?  23  based on: World Directory of Medical Schools https://www.wdoms.org/ | __________________________________ |
| How many Medical Universities or Academies have Chairs or Departments of Family Medicine or General Practice? | __________________________________ |
| How many Chairs or Departments of Family Medicine are chaired by FPs/GPs with a professor title/position? | __________________________________  (FPs/GPs - Family Physicians/General Practitioners) |

Is Family Medicine/General Practice a part of the

Yes

undergraduate medical curriculum?

No

You stated Family Medicine/General Practice is a part

mandatory

of the undergraduate medical curriculum.

facultative (elective)

If so, is it mandatory or facultative (elevtive)?

You stated Family Medicine/General Practice is a

facultative/elective part of the undergraduate medical

__________________________________

curriculum.

If so, how many medical faculties teach it? If you do

not know, please write "?"

In the undergraduate medical curriculum, is there a

Yes

minimum number of teaching hours of Family

No

Medicine/General Practice?

You stated there is a minimum number of teaching hours

of Family Medicine/General Practice?

__________________________________

If so, how many hours is this minimum?

Are there any quality measures or requirements for

no, not at all

FM/GP teaching practices?

yes, but not applied in practice

yes, and they are applied in practice, but they

are not obligatory

yes, they are obligatory

(

FM/GP - family medicine/general practice

)

What is the body/authority which is responsible for

universities/medical faculties

organization of specialty (vocational) training in

separate postgraduate (vocational) training centers

FM/GP. Please, choose all apllicable.

hospitals

FM/GP facilities (practicies, health care centers

etc.)

FM/GP associations/colleges

not applicable (there is no separate FM/GP medical

specialty)

(

FM/GP - family medicine/general practice

)

| What is the overall duration of specialist |  |
| --- | --- |
| (vocational) training in FM/GP in your country (in | __________________________________ |
| months)? | (FM/GP - family medicine/general practice) |

What is the duration of specialist (vocational) training in FM/GP in primary care setting (in months)? __________________________________

)

FM/GP - family medicine/general practice

(

Are the residents (trainees) during their FM/GP

not salaried (volontariate)

trainig salaried?

salaried by the trainers (and/or FM/GP practiecies)

Please choose all applicable

salaried by the state/govenmental institurions

salaried by local authorities

salaried by health insurance companies

salaried by hospitals

)

(

FM/GP - family medicine/general practice

Is there any formal exam at the end of specialty

Yes

(

vocational) FM/GP training?

No

(

FM/GP - family medicine/general practice

)

You stated there is a formal exam at the end of FM/GP

multiple choice test (MCQ)

specialty (vocational) training. What is is it

written (other than MCQ)

consisting of?

oral

practical

other

(

FM/GP - family medicine/general practice

)

Are there in your country any courses for FM/GP

Yes

teachers available (e.g. Leonardo EURACT)?

No

(

FM/GP - family medicine/general practice; EURACT

-

European Academy of Teachers in General

Practice/Family Medicine)

| You stated there are courses for FM/GP teachers |  |
| --- | --- |
| available in your country. | __________________________________ |

If so, how many physicians completed them so far?

(

FM/GP - family medicine/general practice

)

Are there any incentives to chooce specialty

better professional perspectives

(

vocational) training in FM/GP?

higher salary (compared to other vocational

Please, choose all applicable.

trainings)

higher than in other specialties income in future

easy access to specialty training

other (please describe briefly)

no special incentives

(

FM/GP - family medicine/general practice

)

| What was the number of trainees starting the |  |
| --- | --- |
| postgraduate specialty (vocational) training program | __________________________________ |
| in FM/GP in the most recent year | (FM/GP - family medicine/general practice) |

Is there any formal recertification procedure of yes, obligatory Family Physicians/General Practitioners in your yes, volontary

country? no, such a procedure does not exist

Has EURACT Educational Agenda been translated and/or yes, transated and implemented

implemented in your country? yes, translated but not implemented

no, not translated but implemented no, neither translated nor implemented

(EURACT - European Academy of Teachers in General Practice)

**Reserarch and Scientific Activity**

Are there specific postgraduate (Ph.D.) programs in

Yes

FM/GP available?

No

(

FM/GP - family medicine/general practice

)

Do scientific organizations (e.g. college,

Yes

association) of FPs/GPs exist in your country?

No

(

FPs/GPs - family physicians/general practitioners

)

You stated, there is (are) scientific organization(s)

of FPs/GPs in your country. __________________________________

If so, how many? (FPs/GPs - family physicians/general practitioners)

Are there any scientific peer-reviewed journals on

Yes

FM/GP in your country?

No

(

FM/GP - family medicine/general practice

)

You stated there is at least one scientific

peer-reviewed journal on Family Medicine/General

__________________________________

Practice in your country? If so, how many?

Are there scientific FM/GP conferences held at the

Yes

country/national level?

No

| please list all of them and name them  You stated there is at least one country/national | __________________________________________  (FM/GP - family medicine/general practice) |
| --- | --- |
| research network in Primary Care/FM/GP. | __________________________________ |
| How many FM/GP practicies/health care centers participate in them? | (FM/GP - family medicine/general practice) |

(please answer "Yes" also in case it had been replaced (FM/GP - family medicine/general practice)

with a virtual event during COVID-19 pandemic)

You stated there is at least one scientific FM/GP

twice per year or more often

conference held at the country/national level?

annualy

If so, how frequent they are organized?

biannually

every third year or less frequent

there is no regular pattern

(

FM/GP - family medicine/general practice

)

Is there in your country any special/scpecific

Yes

fund/granting program dedicated to support research

No

activies in FM/GP?

I do not know

(

FM/GP - family medicine/general practice

)

You stated there is at least one scpecific

| fund/granting program dedicated to support research in |  |
| --- | --- |
| FM/GP. | __________________________________________ |
| If so, please name it/them and decribe briefly | (FM/GP - family medicine/general practice) |

Is there any country/national research network in

Yes

Primary Care/FM/GP?

No

(

FM/GP - family medicine/general practice

)

You stated there is at least one country/national

research network in Primary Care/FM/GP.
