# Supplementary material for: How far on the road? The role of family medicine/general practice in 10 Central and Eastern European countries: A mixed-method study
Source: Eur J Gen Pract. 2025 Dec 17;31(1):2594292. doi: 10.1080/13814788.2025.2594292 (PMC12713223; doi:10.1080/13814788.2025.2594292)
Supplement: Supplemental Material [file IGEN_A_2594292_SM2077.zip › IGEN_A_2594292_suppl_data/ejgp-2025-0118-File007.docx]

Supplemental Material 3. Predominant or frequent Organisational forms of primary care services

|  | Single-handed (solo) | Single-handed (solo) as part of chains  or cooperatives | Small group practices of less than 6 FPs/GPs | Larger group practices/health centers with 6 or more FPs/GPs | Health centers with FPs/GPs and other specialists |
| --- | --- | --- | --- | --- | --- |
| CZ | 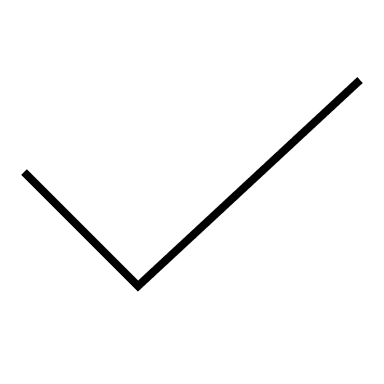 |  |  |  |  |
| EE | 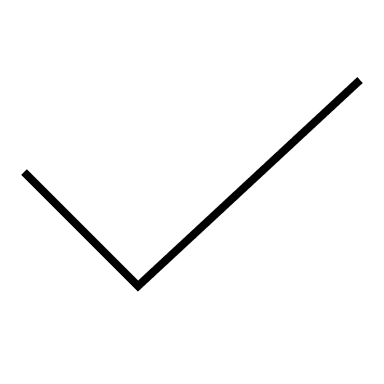 |  | 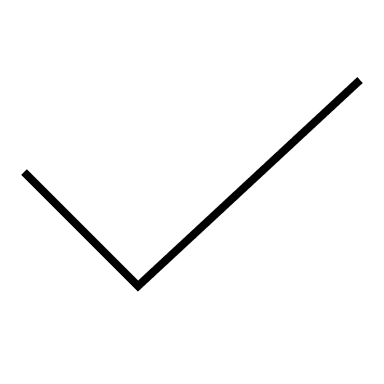 |  | 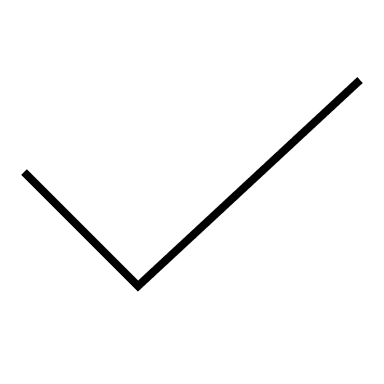 |
| HR | 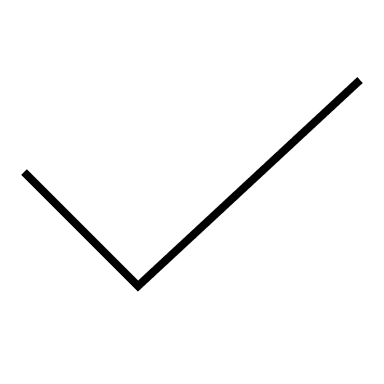 |  |  |  | 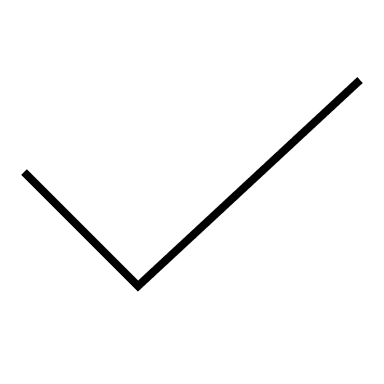 |
| ME |  |  |  | 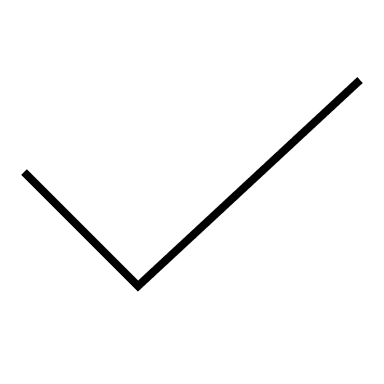 | 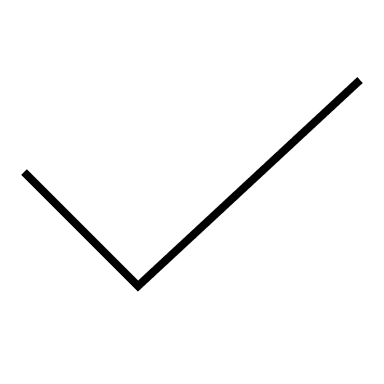 |
| MK | 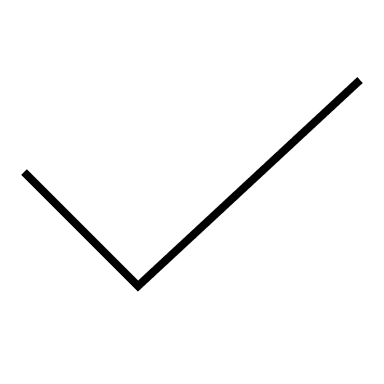 |  |  |  |  |
| PL | 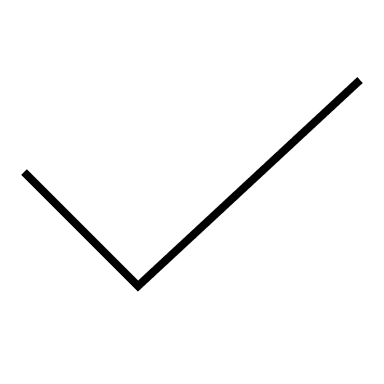 |  | 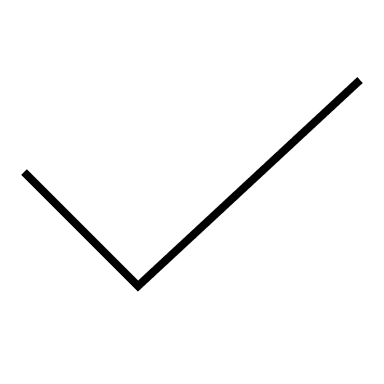 | 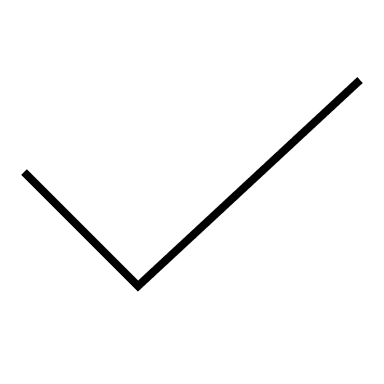 | 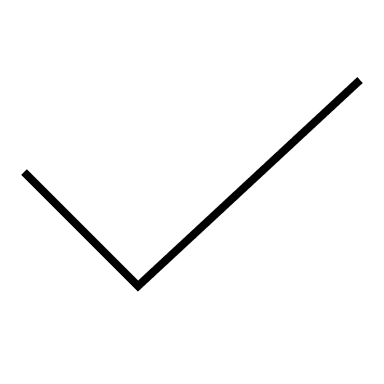 |
| RO | 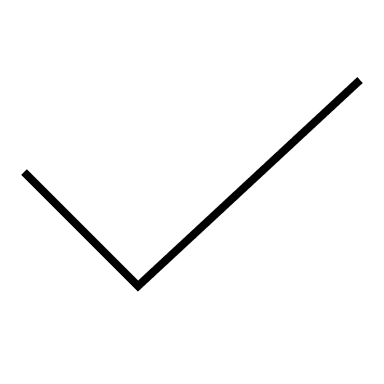 |  |  |  |  |
| RS |  | 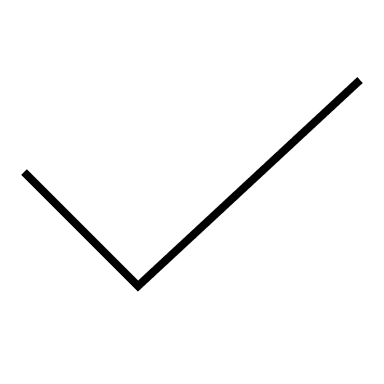 |  | 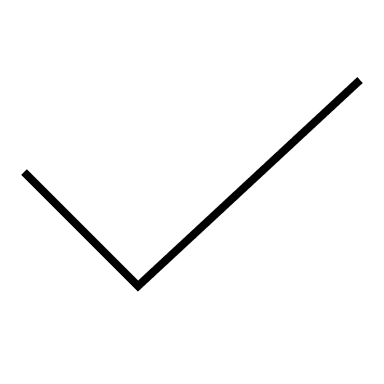 | 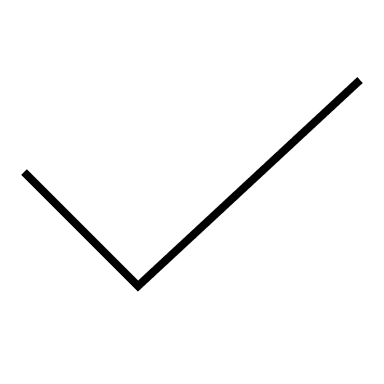 |
| SK | 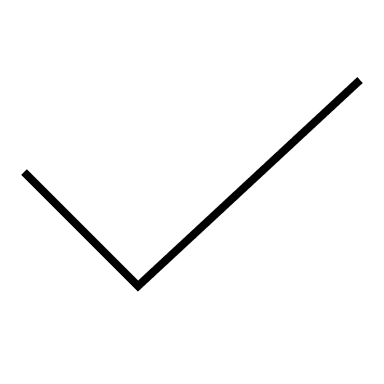 |  |  |  |  |
| SI | 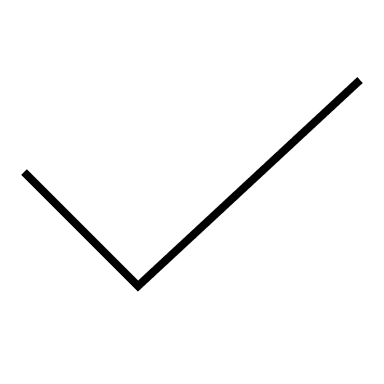 | 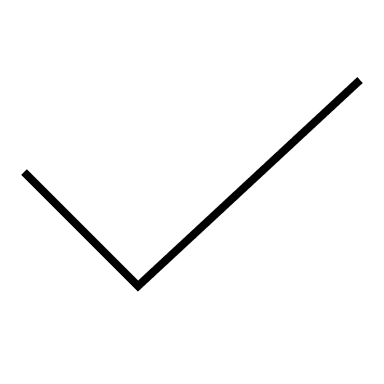 |  | 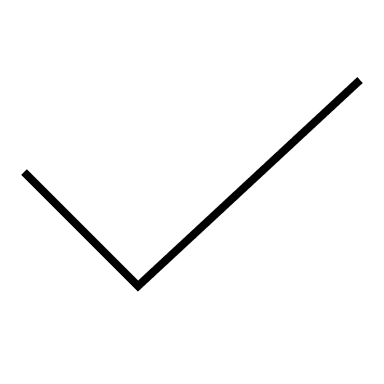 | 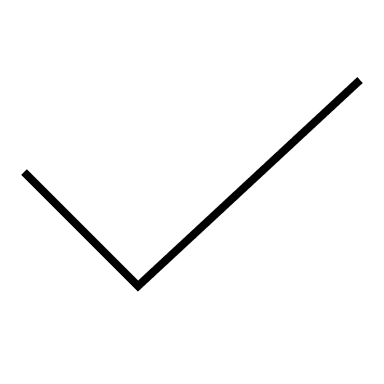 |
| CZ – Czech Republic, EE – Estonia, HR – Croatia, ME – Montenegro, MK – North Macedonia, PL – Poland, RO – Romania, RS – Serbia, SK – Slovakia, SI – Slovenia  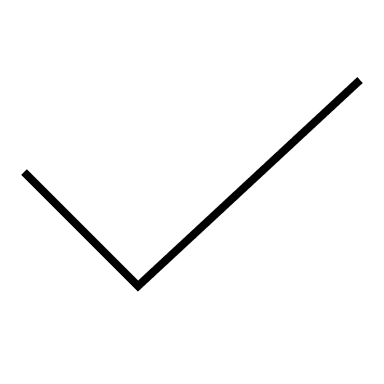 predominant or frequent; FPs/GPs – family physicians/general practitioners | | | | | |
