# Supplementary material for: How far on the road? The role of family medicine/general practice in 10 Central and Eastern European countries: A mixed-method study
Source: Eur J Gen Pract. 2025 Dec 17;31(1):2594292. doi: 10.1080/13814788.2025.2594292 (PMC12713223; doi:10.1080/13814788.2025.2594292)
Supplement: Supplemental Material [file IGEN_A_2594292_SM2077.zip › IGEN_A_2594292_suppl_data/ejgp-2025-0118-File008.docx]

Supplemental Material 4. Regulations of daily working hours of Family Physicians/General Practitioners

| Regulations of daily working hours of Family Physicians/General Practitioners | | | | |
| --- | --- | --- | --- | --- |
|  | Are daily  working  hours  regulated? | hrs/day | hrs/day in direct contact with patients | Comments |
| CZ | Yes | - | - | Practices are available Mon-Fri, number of working and opening hours based on the agreement with insurance companies, required ≥25hrs of consultations/week |
| EE | Yes | 8 | 4 | 40 hrs/week, ≥20hrs of consultations/week |
| HR | Yes | 7.5 | 6 – 7.5 | 7.5 hrs/day (Mon-Fri) and 2 Sat shifts/month (5 hrs each). Regulated by contract with an insurance company. |
| ME | Yes | 7 | 7 | ≥35 hrs/week |
| MK | Yes | 8 | 8 | 8 hours/day (Mon-Fri) |
| PL | No | - | - | Working hours of physicians are not regulated, but PC facilities must be accessible 10 hrs/day (Mon-Fri, 8-18) |
| RO | Yes | 7 | 7 | Each office contracts 35 hours/week or more (7 hours/day: 5 for the office and 2 for home visits), which can be increased to 8-9/day (on agreement with the insurance company). |
| RS | Yes | 8 | 7-8 | 7 hrs on weekdays and on duty on weekends (regulated by Healthcare law, Health Insurance Law, and Employment contract) |
| SK | Yes |  |  | Obligation to provide care ≥35 hrs/week; at least 2x/week ≥3 p.m. |
| SI | Yes | 8 | 7 | 8 hrs/day, ≤48/week; ≤24 hrs in one cluster |
| CZ – Czech Republic, EE – Estonia, HR – Croatia, ME – Montenegro, MK – North Macedonia, PL – Poland, RO – Romania, RS – Serbia, SK – Slovakia, SI – Slovenia  PC – primary healthcare | | | | |
