# Supplementary material for: How far on the road? The role of family medicine/general practice in 10 Central and Eastern European countries: A mixed-method study
Source: Eur J Gen Pract. 2025 Dec 17;31(1):2594292. doi: 10.1080/13814788.2025.2594292 (PMC12713223; doi:10.1080/13814788.2025.2594292)
Supplement: Supplemental Material [file IGEN_A_2594292_SM2077.zip › IGEN_A_2594292_suppl_data/ejgp-2025-0118-File009.docx]

**Supplemental Materials 5: Types of out-of-hours care service providers**

| **Type of service** | **Emergency Services** | **Hospital-Based Centres** | **Rota of Duties (Practices/Physicians)** | **Specialized Companies** |
| --- | --- | --- | --- | --- |
| **Country** | | | | |
| **Czech Republic** | ✓ | ✓ |  | ✓ |
| **Estonia** | ✓ | ✓ |  |  |
| **Croatia** | ✓ |  |  |  |
| **Montenegro** | ✓ |  |  |  |
| **North Macedonia** | ✓ | ✓ |  |  |
| **Serbia** | ✓ |  | ✓ |  |
| **Slovakia** | ✓ | ✓ |  | ✓ |
| **Slovenia** | ✓ | ✓ | ✓ |  |
| **Poland** |  | ✓ |  | ✓ |
| **Romania** |  |  | ✓ |  |
