# Supplementary material for: How far on the road? The role of family medicine/general practice in 10 Central and Eastern European countries: A mixed-method study
Source: Eur J Gen Pract. 2025 Dec 17;31(1):2594292. doi: 10.1080/13814788.2025.2594292 (PMC12713223; doi:10.1080/13814788.2025.2594292)
Supplement: Supplemental Material [file IGEN_A_2594292_SM2077.zip › IGEN_A_2594292_suppl_data/ejgp-2025-0118-File010.docx]

Supplemental Material 6. Comparisons of selected areas of FATMEE and FATMEE-2 results

| **FATMEE and FATMEE-2 comparison by country (2012 vs 2024)** | | | |
| --- | --- | --- | --- |
| **Legal Framework and Recognition** | | | |
| **Country** | **2012 Status** | **2024 Status** | **Comments** |
| Czech Republic | Legal recognition established | Parliamentary acts/ministerial decrees | Stable framework |
| Estonia | Legal recognition with strategic documents | Comprehensive legal framework | Enhanced regulation |
| Croatia | Legal recognition with development strategy | Parliamentary acts with competency descriptions | More detailed framework |
| Poland | Legal recognition established | Parliamentary acts with government regulation | Enhanced regulation |
| Romania | Legal recognition present | Parliamentary acts with government regulation | Enhanced regulation |
| Slovakia | Legal recognition established | Parliamentary acts with clear structure | Enhanced regulatory clarity |
| Slovenia | Legal recognition with strategic approach | Comprehensive parliamentary framework | Robust legal foundation |
| **Primary Care Organization Model** | | | |
| **Country** | **2012 Model** | **2024 Model** | **Comments** |
| Czech Republic | Mixed specialties (pediatricians, gynecologists) | Mixed specialties (unchanged) | No structural evolution |
| Estonia | Exclusive FM/GP model | Exclusive FM/GP model | Maintained exclusivity |
| Croatia | Mixed specialties with health centers | Mixed specialties (limited change) | No structural evolution |
| Poland | Mixed with 2017 transition deadline | Mixed (deadline missed, subcontractors allowed) | No structural evolution, failed transition |
| Romania | FM/GP exclusive model | FM/GP exclusive model | Maintained exclusivity |
| Slovakia | Mixed specialties (pediatricians involved) | Mixed specialties (unchanged) | No structural change |
| Slovenia | Health center model with mixed elements | Health center model with mixed elements | No structural change |
| **Employment and Working Conditions** | | | |
| **Country** | **2012 Status** | **2024 Status** | **Comments** |
| Czech Republic | Independent contractors, 25hrs/week | Independent contractors, 25hrs/week | Unique low-hour model persists |
| Estonia | Independent contractors with regulations | Enhanced contractor model with refined rules | Improved regulatory framework |
| Croatia | State employees with regulated hours | Mixed employment, 7.5hrs/day regulated | More employment options |
| Poland | Mixed employment, facility accessibility rules (50 hrs/week) | No physician hour regulation, facility accessibility rules (50 hrs/week) | No change |
| Romania | Basic employment framework | 7hrs/day regulated (5 office + 2 home visits) | Detailed hour specifications |
| Slovakia | Independent contractors | Independent contractors with service obligations | Stable contractor model |
| Slovenia | Mixed employment options | Enhanced mixed model with cluster arrangements | Advanced organizational model |
| **Payment Systems** | | | |
| **Country** | **2012 System** | **2024 System** | **Comments** |
| Czech Republic | Basic capitation | Capitation + FFS + PFP | Added multiple payment streams |
| Estonia | Capitation with adjustments | Sophisticated capitation with age/chronic disease weights | Highly refined system |
| Croatia | Mixed capitation system | Capitation + performance elements | Added performance incentives |
| Poland | Mixed payment system | Weighted capitation + FFS + PFP | Multiple payment components |
| Romania | Basic capitation | Mixed system with FFS components | Added service-based payments |
| Slovakia | Capitation-based | Capitation + FFS | Added fee-for-service |
| Slovenia | Capitation system | Weighted capitation + FFS + PFP | Added multiple payment streams |
| **Technology Adoption** | | | |
| **Country** | **2012 Status** | **2024 Status** | **Comments** |
| Czech Republic | Mixed EMR/paper | Advanced EMR integration + telemedicine | Major technological change |
| Estonia | Leading EMR adoption | Universal EMR + comprehensive e-health | Maintained technology change |
| Croatia | Limited EMR usage | Universal EMR + telemedicine | Major technological change |
| Poland | Traditional paper records | Mixed EMR/paper | Major technological change |
| Romania | Paper-based systems | Increasing EMR adoption | Ongoing modernization |
| Slovakia | Limited EMR | Widespread EMR + comprehensive systems | Major technological change |
| Slovenia | Moderate EMR adoption | Universal EMR + digital integration | Major technological change |
| **Service Scope and Gatekeeping** | | | |
| **Country** | **2012 Scope** | **2024 Scope** | **Comments** |
| Czech Republic | Basic primary care services | Expanded services + occupational medicine | Added service components |
| Estonia | Comprehensive primary care | Maintained comprehensive scope | Stable service range |
| Croatia | Comprehensive with total gatekeeping | Enhanced services + group sessions + gatekeeping | Added services |
| Poland | Basic services with partial gatekeeping | Standard services with partial gatekeeping | Limited scope evolution |
| Romania | Standard primary care services | Standard services (limited expansion) | Limited scope evolution |
| Slovakia | Standard services with partial gatekeeping | Standard services (unchanged) | No service expansion |
| Slovenia | Comprehensive health center services | Enhanced integrated services + group sessions | Added services |
| FFS – fee-for-service PFP – pay-for-performance | | | |
